# Metal oxide-resistive memory using graphene-edge electrodes

**DOI:** 10.1038/ncomms9407

**Published:** 2015-09-25

**Authors:** Seunghyun Lee, Joon Sohn, Zizhen Jiang, Hong-Yu Chen, H.-S. Philip Wong

**Affiliations:** 1 Department of Electrical Engineering and Stanford SystemX Alliance, Stanford University, Stanford, California 94305, USA

## Abstract

The emerging paradigm of ‘abundant-data' computing requires real-time analytics on enormous quantities of data collected by a mushrooming network of sensors. Todays computing technology, however, cannot scale to satisfy such big data applications with the required throughput and energy efficiency. The next technology frontier will be monolithically integrated chips with three-dimensionally interleaved memory and logic for unprecedented data bandwidth with reduced energy consumption. In this work, we exploit the atomically thin nature of the graphene edge to assemble a resistive memory (∼3 Å thick) stacked in a vertical three-dimensional structure. We report some of the lowest power and energy consumption among the emerging non-volatile memories due to an extremely thin electrode with unique properties, low programming voltages, and low current. Circuit analysis of the three-dimensional architecture using experimentally measured device properties show higher storage potential for graphene devices compared that of metal based devices.

## Abstract (web summary)

Increasing memory performance and density will require new breakthroughs in atomic-scale technology and three-dimensional device architectures. Here, the authors demonstrate a memory just 3 Å thick that can be stacked by exploiting the atomically thin edge of monolayer graphene.

## Graphical abstract

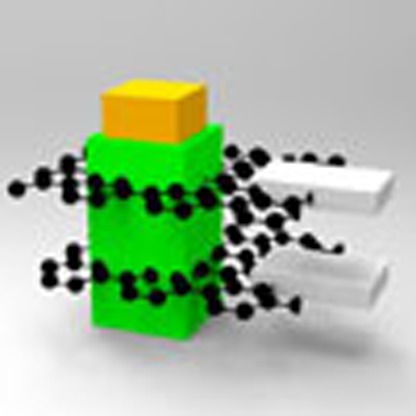

The rapid adoption of non-volatile memory technology such as Flash^1^ has enabled a revolution in today's mobile computing. To date, the ever-increasing demand for higher density has so far been met through the development of multilevel storage cells and smart peripheral control circuitry that hides the inadequacies and imperfections of the memory cell^1^. However, the diminishing amount of stored charges and the increase in bit error rates that accompany feature-size scaling impose significant challenges for the future^2^. Further gains in memory performance and device density will require new breakthroughs in both atomic-scale technology and bit-cost-effective three-dimensional (3D) device architectures^2^,^3^.

Resistive random access memories (RRAM) based on metal oxide have shown considerable promise as a possible successor to Flash because of better endurance, retention, speed, lower programming voltages and a higher device density^3^,^4^,^5^. These devices also use material sets and fabrication temperatures that are compatible with today's silicon technology^3^,^4^, and offer the opportunity for future monolithic 3D integration with logic computation units.

Graphene, an atomically thin crystal lattice of carbon atoms, is known for its unique electronic properties^6^. Both graphene and graphene oxides have been used in various memory devices, including RRAM^7^,^8^,^9^,^10^,^11^, ferroelectric memory^12^ and Flash memories^13^ as electrodes and oxides.

In this work, the atomically thin (∼3 Å thick) edge of monolayer graphene was actively used as a SET electrode to form an atomically thin memory structure. We investigate the low energy consumption and the stacking potential of the device in a 3D architecture that is amenable to large scale manufacturing.

## Results

### Device structure in a 3D vertical cross-point architecture

**Figure 1:**
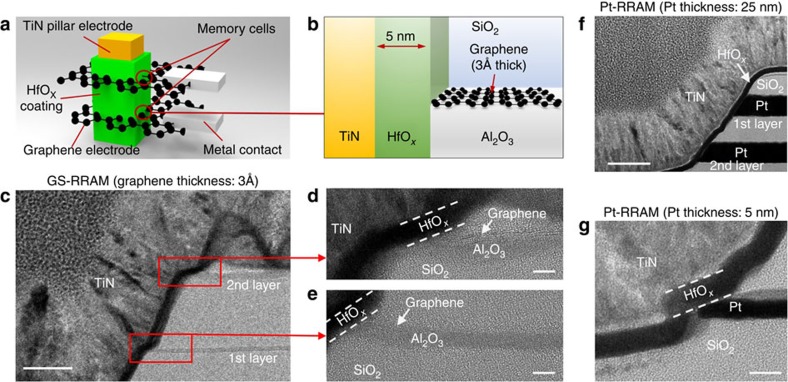
Structure of grapheme-based and Pt-based RRAM in a vertical 3D cross-point architecture. (**a**) An illustration of graphene-based RRAM in a vertical cross-point architecture. The RRAM cells are formed at the intersections of the TiN pillar electrode and the graphene plane electrode. The resistive switching HfO_*x*_ layer surrounds the TiN pillar electrode and is also in contact with the graphene plane electrode. (**b**) A schematic cross-section of the graphene-based RRAM. (**c**) High-resolution TEM image (details in Methods section) of the two-stack GS-RRAM structure. The RRAM memory elements are highlighted in red. Scale bar, 40 nm. (**d**,**e**) First and second layer of GS-RRAM with graphene on top of the Al_2_O_3_ layer. Scale bars, 5 nm. (**f**,**g**) TEM image of the two-stack Pt-based RRAM from previous work^14^,^15^. Scale bars, 40 nm (**f**) and 5 nm (**g**).

Two layers of graphene RRAMs (GS-RRAM, GS stands for graphene SET electrode) were stacked to build a 3D vertical cross-point architecture as illustrated in Fig. 1a,b. In the figures, the TiN electrode, the HfO_*x*_ layer and the graphene electrode are depicted in yellow, green and black, respectively. The fabrication process is explained in Supplementary Figs 1 and 2. A transmission electron microscope (TEM) image of the device's cross-section is presented in Fig. 1c–e. The graphene edge contacting the memory element (HfO_*x*_) is highlighted in red. We also fabricated RRAMs based on platinum electrodes (Pt-RRAM) as control devices. The Pt-RRAM (Fig. 1f,g), which was reported previously^14^,^15^, has the same 3D structure as the GS-RRAM.

Such 3D architectures are part of an ongoing drive in the research community to adopt a bit-cost-effective architecture^1^,^2^,^14^,^15^,^16^,^17^ with storage densities surpassing that of Flash technology (Supplementary Note 1). From past experimental results^16^, the density of a 3D vertical RRAM array is known to be mainly limited by the sheet resistance and the layer thickness of the plane electrode, and not so much by the lithographic half-pitch, as it is in two-dimensional (2D) architectures. This is due to the limitation of the pillar electrode resistance and the non-vertical etching angle resulting from trench etching through metal planes^16^. Graphene's sheet resistance per thickness (125 Ω per square at a monolayer thickness of 3 Å when doped^18^) is significantly lower than that of any metal. All metal films are known to exhibit a steep exponential increase in sheet resistance as the thickness falls below 5 nm (ref. 15). Graphene is also significantly easier to etch vertically than metal during pillar formation. Using a well-accepted reliability projection^15^—assuming programming voltage of 3 V, SiO_2_ thickness of 6 nm, half-pitch of 22 nm and 1° of etch angle improvement—a maximum of 200 stacks will be possible for GS-RRAM as compared with the 60 stacks possible with conventional bulk-metal-based 3D RRAM (Supplementary Table 1).

In both of our RRAM structures, the conductive filaments of oxygen vacancies form at the oxide (HfO_*x*_) similar to conventional metal oxide-resistive memories. The number and the size of the conducting filament (CF) paths determine the two resistance states of the RRAM: the high resistance state (HRS) and the low resistance state (LRS). In the Pt-RRAM structure (Fig. 1g), TiN is used as the SET electrode as in most conventional devices with TiN-oxide-Pt structures^4^,^14^. In the GS-RRAM structure (Fig. 1a–e), however, the graphene electrode is used as the SET electrode to store (SET) and release (RESET) the oxygen ions during the programming process. This is fundamentally different from our previous work^19^ on GS-RRAM where the TiN electrode was the SET electrode. The application of graphene as the SET electrode led to power consumption 120 times lower in this work compared with the previous work^19^.

### The device characteristics of GS-RRAM

**Figure 2:**
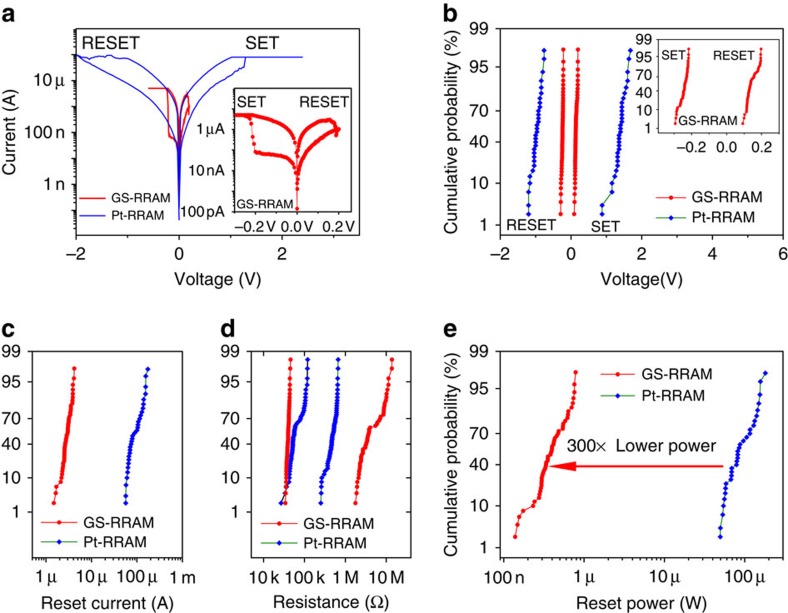
The device characteristics of GS-RRAM compared with Pt-RRAM and other emerging memory devices. (**a**) Typical d.c. I–V switching characteristics of GS-RRAM and Pt-RRAM. For Pt-RRAM, SET process is observed when positive voltage is applied to TiN. For GS-RRAM, SET process is observed when positive voltage is applied to graphene. The SET compliances for GS-RRAM and Pt-RRAM are 5 and 80 μA, respectively, for optimum conditions. A magnified plot of GS-RRAM is shown as inset. (**b**) The SET and RESET voltage distribution of GS-RRAM and Pt-RRAM after 50 cycles of switching. The SET/RESET voltages of GS-RRAM are noticeably lower (inset). (**c**) Reset current distribution of GS-RRAM and Pt-RRAM after 50 cycles. GS-RRAM exhibit much lower reset current compared with Pt-RRAM. (**d**) Resistance distribution after 50 cycles for GS-RRAM and Pt-RRAM at 0.1 V. Larger memory windows are observed for GS-RRAM compared with Pt-RRAM. (**e**) Reset power distribution of GS-RRAM and Pt-RRAM. The power consumption of GS-RRAM is 300 times lower than that of Pt-RRAM. This is from the combined effect of lower programming voltages and currents.

A comparison of the typical SET/RESET switching cycle of the GS-RRAM with the Pt-RRAM is shown in Fig. 2a (inset: magnified view of GS-RRAM plot). The SET programming is achieved by applying a positive voltage to the TiN electrode in the Pt-RRAM and a negative voltage to the TiN electrode in the GS-RRAM. The SET/RESET voltage and the RESET current distribution of GS-RRAM and Pt-RRAM after 50 cycles of switching are shown in Fig. 2b,c (the values for Pt-RRAM are in agreement with the refs 14, 15). Importantly, the SET/RESET voltages and the RESET currents of GS-RRAM are considerably lower than those of Pt-RRAM. The resistance distributions of both the HRS and the LRS states at 0.1 V bias after 50 cycles for both devices are shown in Fig. 2d. Even with such low programming voltages and current, the memory window is larger for GS-RRAM compared with Pt-RRAM (Fig. 2d).

The power consumption of an RRAM cell is given by the product of the programming voltages and the currents^4^. Owing to such low SET/RESET voltages and currents, the power consumption of the GS-RRAM is 300 times lower than that of the Pt-RRAM (Fig. 2e). In fact, the power consumption of the GS-RRAM is one of the lowest compared with recent reports on low-power RRAMs (Supplementary Fig. 3). From the pulse-mode endurance test with 500 ns width pulse (see Methods section), the switching energy (switching voltage × current × pulse width=0.2 V × 2.3 μA × 500 ns) was found to be around 230 fJ. We compared this value with the values of other emerging non-volatile memories, including RRAM, conductive bridge RAM, phase change RAM and magnetic RAM in Supplementary Fig. 4, and found the energy consumption to be comparable to the lowest known values.

### The oxygen ion migration and Raman imaging

**Figure 3:**
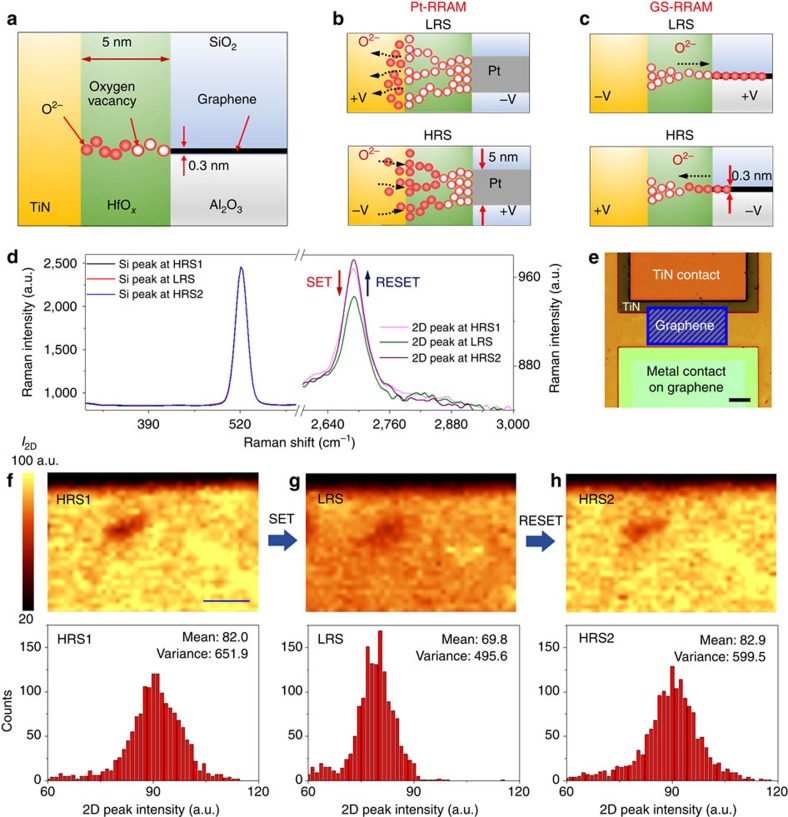
The working mechanism and spatially resolved Raman imaging of oxygen ions in graphene during subsequent SET/RESET process of GS-RRAM. (**a**) Illustrations of the GS-RRAM structure. (**b**) Working mechanism of Pt-RRAM. SET process (oxygen vacancy filament formation) is achieved by applying positive voltage to the TiN electrode. (**c**) Working mechanism of GS-RRAM. The SET process is achieved by applying positive voltage to graphene instead of the TiN electrode. Notice the opposite direction of oxygen ion movement in GS-RRAM compared with Pt-RRAM. (**d**) Changes in the 2D peak intensity as the oxygen is inserted (SET) and extracted (RESET) from the graphene film. The laser intensity was kept constant during the measurements. Notice that the reference silicon peak (520 cm^−1^) is not changing during this transition. (**e**) A microscopic image of the Raman-mapped area highlighted in blue. Scale bar, 15 μm. (**f**–**h**) 2D Raman scanning of the 2D peak intensity in the mapped area before programming (**f**) after oxygen ions are inserted into graphene via SET process (**g**) and after oxygen ions are pulled out from graphene via RESET process (**h**). All three images have the same colour scale for 2D peak intensity, and the laser intensity was kept constant during the measurements (see Methods section). The darker hue is observed for graphene with the oxygen ions in **g**. Scale bar, 10 μm. The statistical distributions of the 2D peak intensity changes are also shown as histograms. Noticeable changes in the median values are observed as the oxygen ions are inserted into and pulled out from the graphene film.

The mechanism behind the low power/energy consumption can only be explained by first understanding the oxygen ion migration during the switching process. Figure 3b and c illustrates the different ways the oxygen ions move and form conductive filaments during the programming process of the Pt-RRAM and the GS-RRAM. For Pt-RRAM, the TiN is the SET electrode and the CFs in the oxide are formed via oxygen migration from HfO_*x*_ to the TiN electrode (Fig. 3b)^4^,^14^.

In a GS-RRAM, however, a negative voltage is applied to the TiN electrode during the SET process, and the oxygen ions move towards the graphene (Fig. 3c). Unlike in conventional metal, there will be an electrical potential gradient in graphene since graphene is relatively more resistive (∼6 kΩ per square) than a common metal. Hence, the oxygen ions will not accumulate at the edge but will migrate horizontally in the graphene and the oxide interface. In our previous work^20^, we have shown how oxygen ions migrate on graphene during the programming process of the RRAM cell by employing Raman spectroscopy (Supplementary Note 2). In this work, the oxygen ion movement was also confirmed by monitoring oxygen dopants in graphene using Raman spectroscopy (Fig. 3d–h , also see Methods section). One of the most pronounced indicators of dopants in graphene is the reduction of 2D peak intensity in a Raman spectrum^20^,^21^. In Fig. 3d,a typical change in the 2D peak (2,670 cm^−1^) intensity is observed for HRS→LRS→HRS transition. During the SET process (that is, HRS→LRS), oxygen ions are inserted into the graphene, doping the film. Consequently, a decrease in the 2D peak intensity is observed. During the RESET process (that is, LRS→ HRS), oxygen ions are pushed back into HfO_*x*_ from the graphene film. This results in an increase in 2D peak intensity. The Raman peak intensity of silicon (520 cm^−1^) and the baseline are plotted in parallel to ensure that the references have not changed during measurement (see Methods section).

The spatially resolved Raman spectroscopy results for the change in 2D peak intensity during the HRS→LRS→HRS transition are shown in Fig. 3f–h, respectively. The blue square in Fig. 3e indicates the Raman-mapped region in the actual device. As the device is switched from HRS to LRS via the SET process, the change in the 2D peak intensity can be readily observed by the contrast difference. The statistical distributions of the changes in 2D peak intensity are also shown as histograms. Noticeable changes in the median values and the s.d. of the 2D peak intensity are observed as the oxygen ions are inserted into and pushed back from the graphene film. This oxygen migration in graphene is also known to be aided by the Joule heating generated during the SET/RESET event^20^,^22^. Experimental studies also suggest that oxygen can be highly mobile in graphene^20^,^22^ and can be used as an oxygen capturing layer^20^,^23^. As indicated in the literature^20^, the oxygen may form a covalent bond with the broken bonds of graphene after the SET process, and the process is reversed during the RESET process (Supplementary Fig. 5).

### The working mechanism

The GS-RRAM offers significantly lower power consumption compared with Pt-RRAM due to three factors: low SET compliance current (Fig. 2a), low RESET current (Fig. 2c) and low programming voltages (Fig. 2b). The Pt-RRAM cannot be operated with such low currents or voltages, and shows severe degradation of the memory window when it is programmed with a lower compliance current (Supplementary Fig. 6).

**Figure 4:**
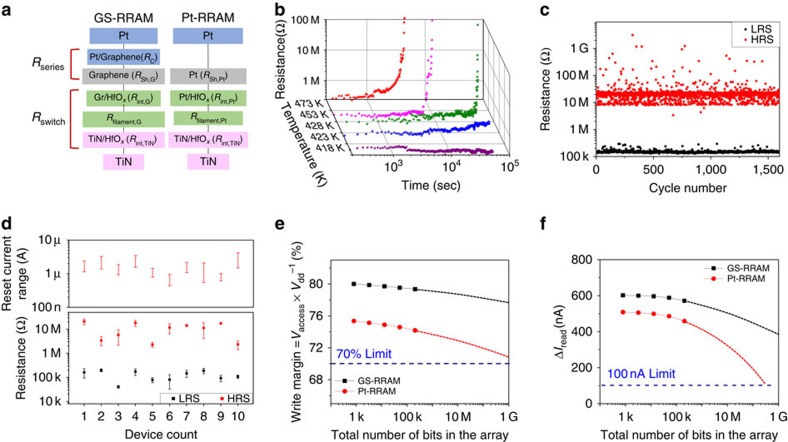
Resistance component breakdown, retention, pulse endurance, device variations and array performance of stacked GS-RRAM. (**a**) Resistance component breakdown of GS-RRAM and Pt-RRAM. In comparison with Pt-RRAM, GS-RRAM has four different resistance components: Pt/graphene contract resistance (*R*_C_), graphene film resistance (*R*_sh,G_), graphene/HfO_*x*_ interface resistance (*R*_int,G_), and the thickness of the conduction filaments (*R*_Filament,G_). (**b**) Temporal evolution of GS-RRAM LRS resistance at temperatures ranging from 418 to 473 K near 0.1 V bias. Elevated temperatures were used in this study to obtain the critical time (that is, filament rupture time) for oxygen migration within a reasonable time frame (see Methods section). (**c**) Pulse endurance test of GS-RRAM. Device switched with over 70 × difference in HRS and LRS, and suffered no read/write disturbance after >1,600 cycles. (**d**) The maximum to minimum reset current distribution (top) and HRS/LRS resistance distribution after 50 cycles (bottom) for 10 randomly chosen GS-RRAMs. The cycle-to-cycle variations are shown as error bars, which represent 1 s.d. for each case. All devices were measured under the SET compliance current of 5 μA. The worst-case scenario still exhibits HRS to LRS ratio exceeding 10 × . (**e**) Write margin comparison of Pt-RRAM with GS-RRAM for a 3D architecture with 200 stacks. (**f**) Read margin comparison between Pt-RRAM and GS-RRAM for a 3D architecture with 200 stacks.

The low SET compliance current in GS-RRAM is possible due to a more resistive HRS and a larger memory window (Fig. 2d) compared with Pt-RRAM. Since the magnitude of the RESET current is directly proportional to the SET compliance current^4^, the low RESET current is also related to these two factors. A systematic breakdown of the resistance components is necessary to understand the differences in LRS/HRS of the two devices (Fig. 4a). Three factors may contribute to the increased resistance of HRS in GS-RRAM compared with Pt-RRAM: the access series resistance *R*_series_ from the graphene sheet compared with the Pt sheet, the difference of the TiN/oxide (*R*_int,TiN_) and graphene/oxide(*R*_int,G_) interface, and the different sizes (Fig. 3b,c) of filamentary conduction paths in HfO_*x*_ (*R*_filament,Pt_ and *R*_filament,G_). From a transmission line measurement (Supplementary Figs 7 and 8), we found that compared to Pt the additional sheet resistance and the contact resistance of graphene contributed little to the total resistance of HRS. On the other hand, the filamentary resistance and the interfacial resistance between materials (graphene, Pt or TiN to HfO_*x*_) dominated the total resistance change during the SET/RESET process.

It is known that in an RRAM structure, the resistance of HRS increases as the inverse of the cell area, roughly following Ohm's law^4^. Specifically, the higher HRS of the GS-RRAM compared with Pt-RRAM is closely related to the tail-end thickness of the CFs in the HRS conditions (Fig. 3b,c bottom panels). Because of the thicker Pt electrode edge compared with the graphene edge, the tail end of the CF will be thicker in the Pt-RRAM compared with the ones in the GS-RRAM. This greater thickness results in the more conductive HRS of Pt-RRAM.

The LRS of these devices are related not only to the size of the filaments but also to the different effects of oxygen in the TiN and the graphene electrodes. The LRS of Pt-RRAM (Fig. 2d) is comparable to that of GS-RRAM, even with larger filaments (Fig. 3b,c, top panel). This is owing to the effect of oxygen in TiN. It is fairly well known that oxygen forms a thin TiO_*x*_N_1−*x*_ film in the TiN layer, which works as a barrier against diffusion and carrier transport^24^. Such a barrier increases the interfacial resistance for Pt-RRAM (*R*_int, TiN_), and the total resistance at LRS becomes comparable to that of GS-RRAM.

The low SET/RESET voltage is related to the thickness of the electrode and the oxygen migration mechanism. After the forming process (Supplementary Fig. 9), the tip of the CF will be near the top electrode (Fig. 3c). The graphene serving as the SET electrode will have a much stronger electric field at the edge compared with the large TiN electrode because graphene is monolayer thick. Therefore, a lower SET voltage will be sufficient to pull the oxygen ions from the oxide. On the other hand, we expect that the lower RESET voltages are attributed to the lower activation energy for oxygen migration in graphene and the absence of a TiO_*x*_N_1−*x*_ diffusion barrier that is typically formed in TiN electrodes. The activation energy of diffusion for oxygen in graphene (0.15–0.8 eV, carrier density dependent)^25^,^26^ is known to be lower than that of TiN (0.95–2.1 eV)^27^. Since the RESET mechanism is closely related to the oxygen diffusion assisted by Joule heating^20^ and its activation energy, the required electrical potential for RESET will be lower for the graphene electrode than for the TiN electrode. The temperature-accelerated LRS retention-time measurement can probe the thermal activation of oxygen ion migration from the graphene to the oxide, as shown in Fig. 4b. From the linear fitting of the Arrhenius plot (Methods section and Supplementary Fig. 10), we estimate the activation energy for oxygen ion migration in graphene to be 0.92 eV, which is lower than the known values for TiN. It is worth noting that the work functions of graphene (4.56 eV) and TiN (4.5 eV) are comparable, and the difference in SET voltages cannot be explained by work function difference alone.

The result of the pulse-mode endurance test in Fig. 4c indicated that the GS-RRAM maintained large memory window (>70 × ) and showed no sign of deterioration after more than 1,600 cycles of switching (Methods section). The yield of the GS-RRAM (88%) was also comparable to that of the Pt-RRAM (92%). The reset current and the HRS/LRS characteristics of 10 randomly chosen GS-RRAM devices are shown in Fig. 4d. We also compare the first and the second layer devices in Supplementary Fig. 11.

### 3D array simulation

The storage density of a cross-point architecture is ultimately limited by the sneak-path leakage in the half-selected and unselected cells^16^,^28^,^29^. During the write operation, the extra voltage drop along the interconnects caused by the leakage current can lead to an insufficient voltage at the selected cell. During the read operation, parasitic conducting paths in unselected cells can degrade the output signal. To systematically investigate how the sneak-path leakage would limit the bit storage capacity of the 3D memory array, a Simulation Program with an Integrated Circuit Emphasis (HSPICE) circuit simulation^16^,^28^,^29^ for the 3D array is performed, using the experimentally measured device properties (see Methods section). Simulations are done using the worst-case data patterns^28^ with the 0.5 × *V* write scheme and the column parallel read scheme^29^. The write margin (*V*_access_ to the *V*_dd_ ratio) and the readout margin (Δ*I*_read_, the current difference between the on and the off state) as a function of total number of bits for the GS-RRAM and Pt-RRAM arrays are simulated under worst-case conditions assuming 200-layer stacks (Fig. 4e,f). The criteria that limit the total number of array bits during write and read operation are set at 70% and 100 nA, respectively. In Fig. 4e,f, we observe that the write/read margin for GS-RRAM is larger and its degradation less pronounced, compared with those of Pt-RRAM, as the arrays become larger. This is a direct consequence of smaller pillar resistance enabled by thinner stacks of the graphene plane electrode with lower sheet resistance. Consequently, a larger array of graphene-based RRAM can be assembled without the adverse sneak-path leakage effect.

## Discussion

In this work, we demonstrated how the unique advantages of a 2D material can be exploited to outperform conventional materials in today's electronic applications. The E-field from the atomically thin edge electrode and the efficient ion storing/transport mechanism of graphene led to significantly lower power consumption. Graphene was also found to be the key enabler for ultra-high-density, bit-cost-effective 3D RRAM arrays. The increased density and the low power consumption of an RRAM structure will enable significant progress in emerging application areas such as energy-efficient abundant-data computing and neuromorphic computing^30^. RRAMs employing various oxides have already been demonstrated for spike-timing-dependent plasticity^30^. A highly integrated electronic synapse network employing low-power graphene memory in a bit-cost-effective 3D architecture will be a significant step towards a highly efficient, next-generation computing system.

## Methods

### High-resolution TEM sample preparation and imaging

The TEM-ready samples were prepared using the *in situ* Focused ion beam lift-out technique on an Dual Beam for Focused Ion Beam/Scanning Electron Microscopy (FEI company, UK). For the imaging, we used an Tecnai TF-20 Field Emission Gun/TEM (FEI company, UK) operated at 200 kV in bright-field TEM mode or high-resolution TEM mode.

### Spatially resolved Raman spectroscopy

The images were taken with constant laser intensity right after the SET and the RESET programming. External perturbation was minimized with an oxide capping layer. For the purpose of Raman measurement, single-stack GS-RRAM (without the second stack) was fabricated and measured to eliminate any effect from the second graphene layer. A WiTec 500 AFM/micro-Raman scanning microscope was used for the 2D Raman raster scanning of graphene. A 532-nm wavelength was used for all measurements. A 30 × 60 μm area was scanned with an integration time of at least 4 s with a 1-μm resolution. Each measurement was conducted in <3 h.

### Extraction of activation energy

The temperature-accelerated LRS retention-time measurement can probe the thermal activation of oxygen ion migration, as shown in Fig. 4b. This will cause the oxygen ions to migrate back to HfO_*x*_, increasing the resistance (that is, RESET) of the RRAM. The kinetics of this process can be described by the Arrhenius law.


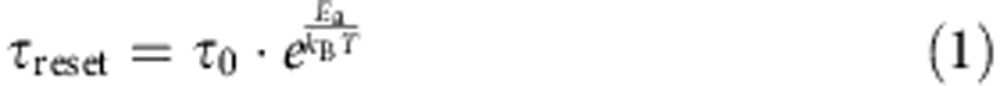


The *τ*_reset_ is the characteristic time for RESET transition, *τ*_0_ is a constant, *k*_B_ is the Boltzmann constant, *E*_a_ is the activation energy barrier and *T* is the absolute temperature. The linear fitting result of retention time in logarithmic scale versus reciprocal temperature provides a good estimation of the activation energy (Supplementary Fig. 10).

The measurements were done on a semi-automated probe system (Cascade Microtech, Summit) with a temperature controller (Temptronic SA166550). All measurements were done inside the test chamber with the nitrogen gas flowing. The set-up was on an anti-vibration table with pneumatic vibration mount. The automated resistance measurement was conducted every 15 s to 3 min with 0.1 V bias using a semiconductor parameter analyser (Agilent 4156C).

### Pulse-mode endurance test

The pulse-mode endurance test was conducted with an Agilent Parameter Analyzer 4155C and an Agilent Pulse generator 81110A connected to a Keithley Switch Matrix 707B. Pulse width was 500 ns with 3 s time delay and ±0.2 V was the read voltage.

### HSPICE simulations on the achievable array size

We adopted the same resistance network and array simulation methodology for the worst-case selected cell of 3D RRAM as in refs 16, 28, 29. The effect of the sneak-path leakage in the achievable array size can be quantified with the write margin (*V*_access_ × *V*_dd_^−1^) and the readout margin (Δ*I*_read_). The definition of *V*_access_ is the voltage across the accessed cell in the resistance network. Δ*I*_read_ is defined as the difference in the current flowing through the read resistor (100 kΩ) when the RRAM cell is either in the HRS or the LRS. The HRS and the LRS values of GS-RRAM and Pt-RRAM were extracted from the experimental results of this work. *V*_dd_, *V*_read_ and *V*_half-bias_ were set at 5, 3.5 and 2 V, respectively. The maximum total bits for an array were determined using these criteria. The sheet resistance of Pt^15^ and doped graphene^18^ was assumed to be 300 Ω per square and 125 Ω per square, respectively. A selector parameter from a published result^31^ was adopted for the simulation. The resistance of the selector was 57.9 MΩ at the half-bias condition and 1 kΩ when it was turned on. During read programming, the selector was turned on and the resistance of the LRS of RRAMs was at least five times larger than the resistance of the selector during read operation. Feature size was 45 nm with a 12-nm selection material layer inserted in the pillar. The diameter of the Cu metal core was 5 nm and the thickness of TiN was 3 nm. The thickness of HfO_*x*_ was 5 nm. Hence, the feature size was 2 × (5+3+12)+5=45 nm.

## Additional information

**How to cite this article:** Lee, S. *et al.* Metal oxide-resistive memory using graphene-edge electrodes. *Nat. Commun.* 6:8407 doi: 10.1038/ncomms9407 (2015).

## Supplementary Material

Supplementary Information Supplementary Figures 1-11, Supplementary Table 1, Supplementary Notes 1-2 and Supplementary References
