# Supplementary material for: Metal oxide-resistive memory using graphene-edge electrodes
Source: Nat Commun. 2015 Sep 25;6:8407. doi: 10.1038/ncomms9407 (PMC4598621; doi:10.1038/ncomms9407)
Supplement: Supplementary Information — Supplementary Figures 1-11, Supplementary Table 1, Supplementary Notes 1-2 and Supplementary References [file ncomms9407-s1.pdf]

Supplementary Figures

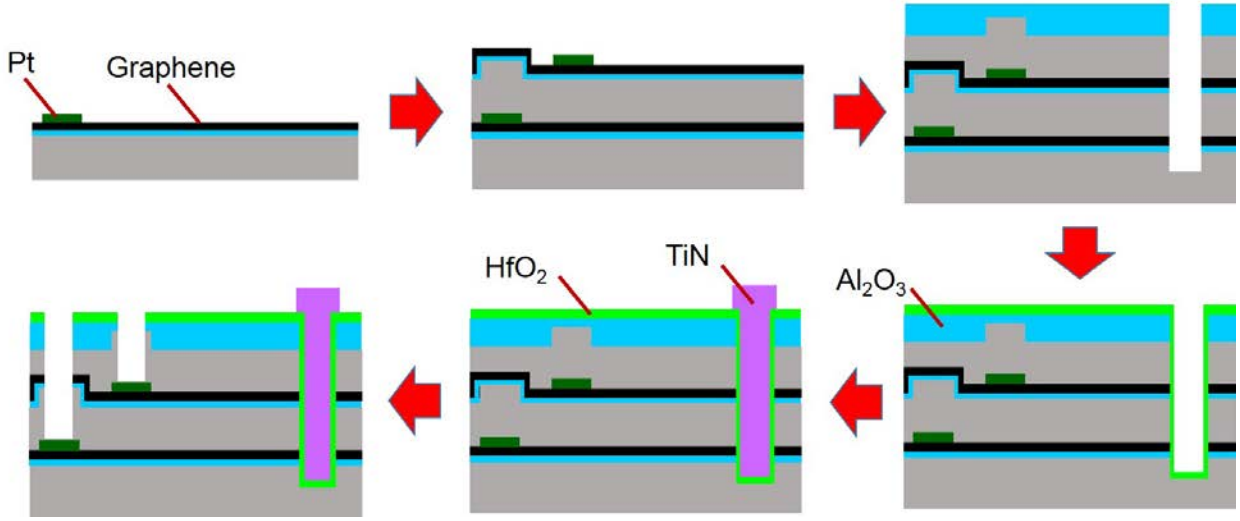

Supplementary Figure 1 Fabrication process. The devices were fabricated via sequential transfer of graphene, metal contact deposition, deep trench etching, HfO_2 deposition (atomic layer deposition) followed by TiN pillar formation (sputtering and lift-off) A thin layer (5nm) of Al_2O_3 was deposited before the graphene transfer process to promote graphene adhesion to the surface. First, single layer graphene is transferred on to a dielectric surface with 5 nm Al_2O_3 and 100 nm of SiO_2. The transfer method is identical to the previous works[1-3]. Monolayer graphene grown on copper foil with chemical vapour deposition method was purchased (Single Layer Graphene on Copper foil: 2 inch × 2inch, Graphene supermarket) and the monolayer quality was confirmed with Raman spectroscopy (Supplementary Figure 2c). Ti/Pt (3 nm/30 nm) layers are deposited by evaporation and patterned by lift-off process. 60 nm of SiO_2 (LPCVD) is deposited. Then these processes are repeated twice for two layers of single layer graphene; 50 nm ALD Al_2O_3 is deposited on the top layer for etch hard mask. A trench is etched down to the bottom SiO_2 layer followed by 5nm of HfO_x (ALD) which is conformally deposited as the active resistive switching layer and 200 nm of TiN electrode is deposited by sputtering and patterned via lift-off. The contacts are opened via dry etching.

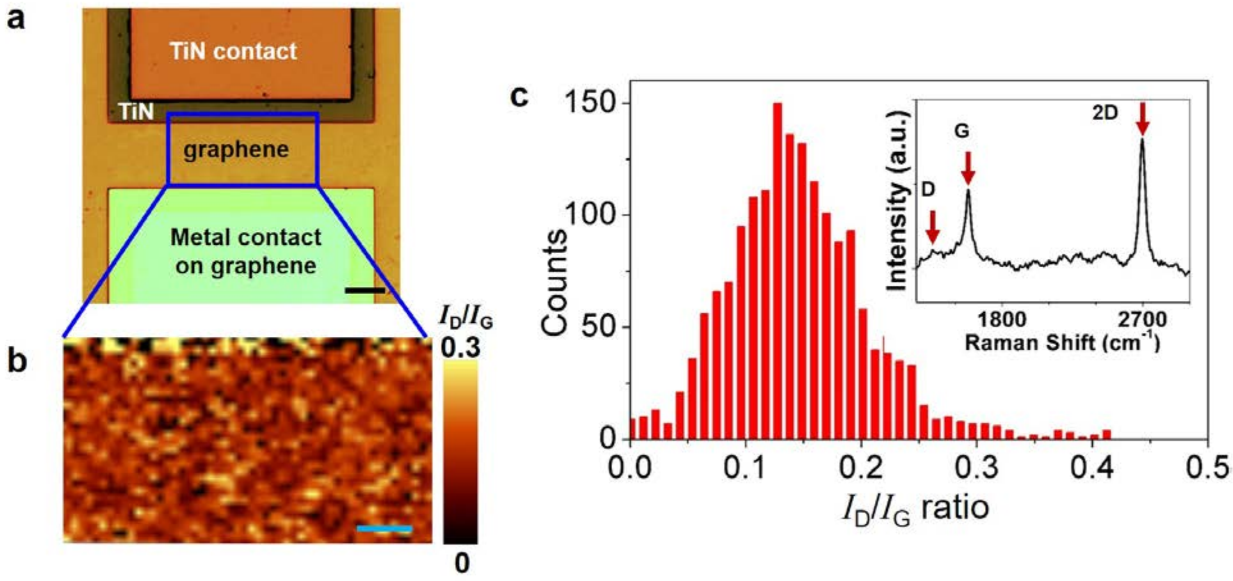

Supplementary Figure 2 Verification of graphene thickness and quality. a, Actual device image. The Raman laser scanned area is highlighted in blue. The scale bar is 15 μm. **b,** A 2D Raman spectra map of D peak to G peak ratio (I_D/I_G) after the complete fabrication process. This ratio is a known indicator of the disorders in graphene films. The I_D/I_G value is limited to approximately 0.1, indicating a low defect density in the film[4]. The inset shows a typical Raman spectrum of monolayer graphene with weak D-peak intensity after the complete fabrication process. Minimized physical disturbance and the low fabrication temperature (<300 °C) were essential to maintaining the high quality graphene. The scale bar is 10μm. **c,** A histogram of I_D/I_G ratio of Supplementary Figure 2b. The median value is 0.12. A typical Raman spectrum of the scanned area is shown as the inset.

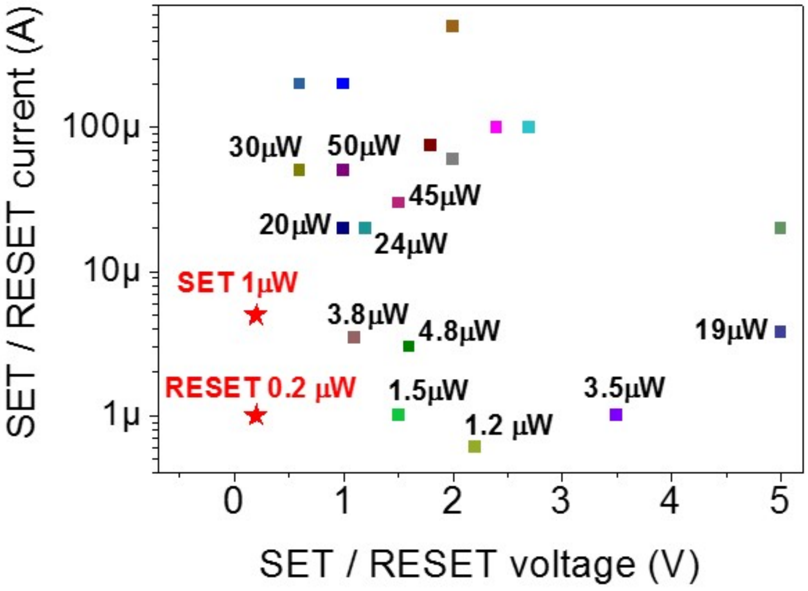

Supplementary Figure 3 Comparison of power consumption. Programming voltages, currents, and power consumptions from the recent reports[5-23] on low power RRAMs were plotted. With one of the lowest SET/RESET voltages ever recorded, the SET and the RESET power consumption of the demonstrated GS-RRAM (shown as red stars above) exhibit extremely low values. From a practical application point of view, the process that consumes the most power (SET or RESET) is plotted for other works, since the larger value determines the power delivery requirements for the chip.

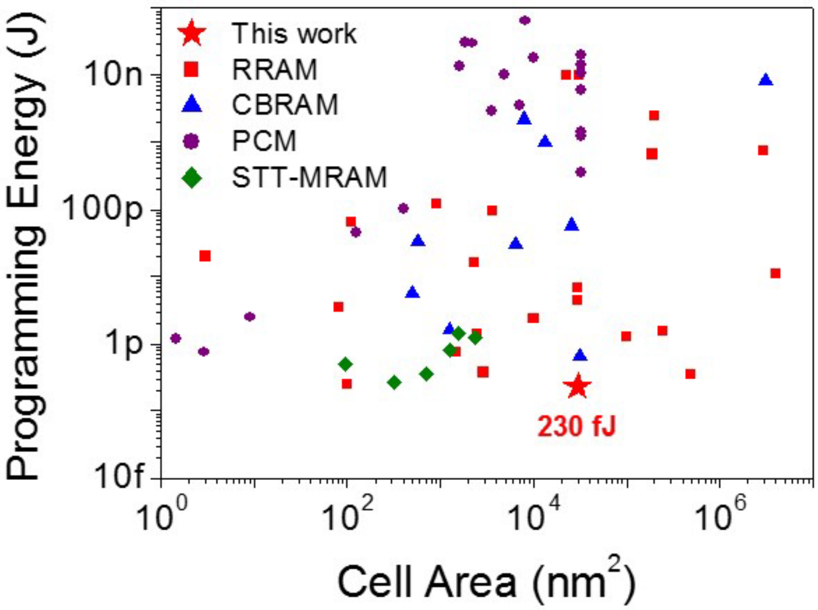

Supplementary Figure 4 Comparison of energy consumption. Comparison of programming energy for GS-RRAM and other emerging non-volatile memories with respect to cell area. The switching energy for GS-RRAM is one of the lowest. RRAM references are [5,11,24-47], CBRAM references are [48-56], PCM references are [57-76], and STT-MRAM references are[77,78], repectively.

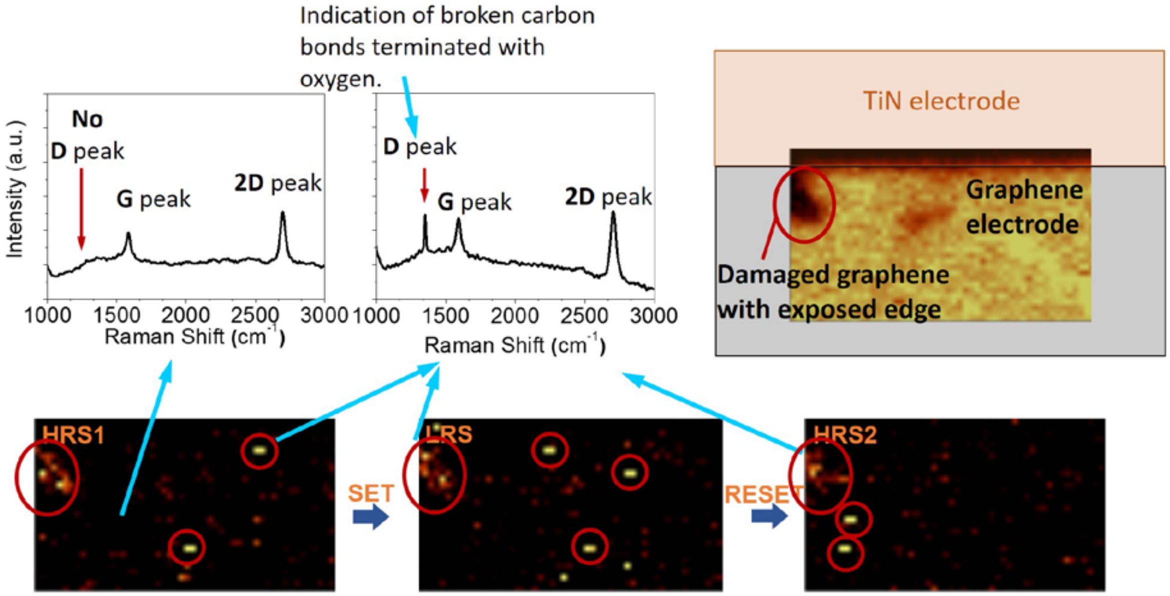

Supplementary Figure 5 Oxygen bonding in the graphene electrode. Although graphene is widely known to be inert, the edge and the broken bonds at the defect sites are more active compared to the basal plane of the graphene sheet. Typical graphene oxide Raman signature is the pronounced D peak[79]. (The intensity of G peak, on the other hand, is associated with the number of graphene layers and this may or may not be related to the graphene oxide.) D peak is also a strong indication of broken carbon bonds (i.e. dislocations, defects) and is pronounced in graphene ribbons with the edges exposed. These broken carbon bonds are more likely to be terminated with oxygen atoms. We specifically found an area in one of devices where the graphene was damaged and the edge was exposed. This edge is composed of broken carbon bonds similar to defects/dislocations at the basal plane and can be detected with the D peak intensity map as shown below in Supplementary Figure 3. An interesting aspect is that the defect region (bright area) highlighted with red circles seems to be created/annihilated (or even shifted) after consecutive SET and RESET process. Several past research results confirm that graphene broken bonds (dislocations) can be created/annihilated and shifted depending on which state is more thermodynamically favorable[80,81]. More importantly, this indicate that these oxygen binding phenomenon is reversible as previous work[82] suggested. As indicated in the reference[82], the oxygen may form a covalent bond at the defect sites of graphene after the SET process and the process is reversed during the RESET process. Another important observation is that the point defects seems to be created and annihilated randomly but at the edge, the bright colored region is pervasive regardless of whether it is after the SET or the RESET process. This may indicate that the edge is always oxidized when it is in contact with the HfO_x. The oxidized edge seems to have little effect on switching endurance of the device. We have switched the device more than 1600 times to observe that the memory function did not degrade (Fig. 4c).

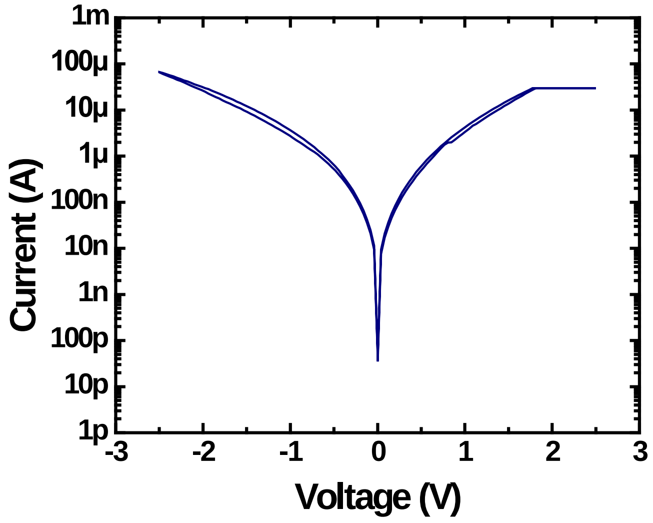

Supplementary Figure 6 Degradation of memory window for Pt-RRAM with 30 μA SET compliance. Pt-RRAM devices with lower SET compliance than 80 μA suffers from memory window degradation as shown in the plot. This is expected since PtRRAM's HRS is significantly more conductive compared to GRRAM due to the larger area of the Pt bottom (passive) electrode.

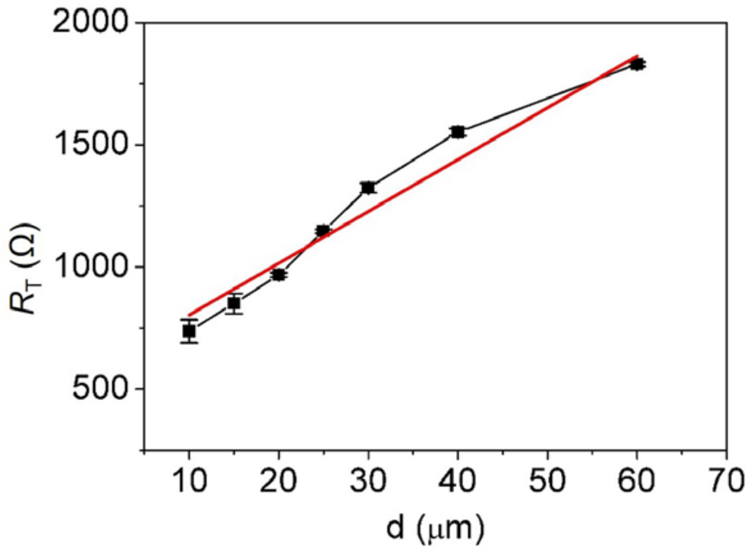

Supplementary Figure 7 Total resistance value from the as-fabricated wafer with circular transmission line test structure[83] as a function of gap distance. From the Y-intercept = $2R_C$ = 591Ω. The corresponding contact resistance R_C between the graphene and the metal (Ti/Pt) contact was found to be 295Ω with specific contact resistance of 9.3Ω·cm. With the slope of 21.2 Ω μm^{-1}, the sheet resistance of graphene ($R_{sh,G}$) is extracted to be 6.7kΩ per square. Pristine, exfoliated graphene without environmental doping is reported to have sheet resistance value of ~6 kΩ per square.[84]. From our I_D/I_G Raman map (Supplementary Figure 2), the defect level was not significant after dielectric deposition (LTO, 300°C). Considering the low D-peak level in our graphene, the resulting $R_{sh,G}$ is in close agreement with that of a pristine graphene that is void of any dopants or defects [85-87]. Since the measurements are done on the as-fabricated wafers, small discrepancies may arise from the process conditions. For Ti/Pt layer (Ti 1 nm/Pt 5 nm), the sheet resistance $R_{sh,Pt}$ was extracted to be 558Ω from 20 TLM measurements. The Pt sheet resistance is also in agreement with the literature [88]. Graphene is approximately ×20 thinner than the Ti/Pt layer and ×12 more resistive, showing slightly superior conductance with similar thicknesses. However, it should be noted that atomically thin metal such as Pt layer tends to form discontinuous island, and a sharp nonlinear increase in sheet resistance is observed as the thickness decreases [88].

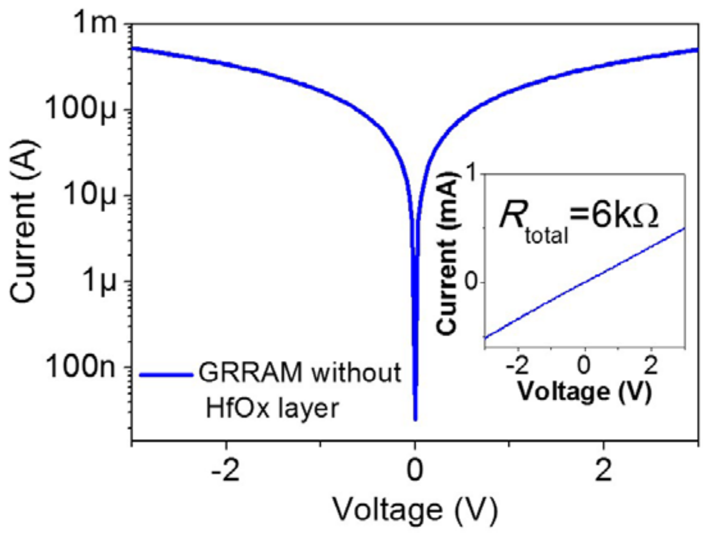

Supplementary Figure 8 The I-V curve of GS-RRAM without the HfOx layer (inset: linear scale). The I-V curve of GS-RRAM without the HfOx layer (inset: linear scale). The total resistance of the GS-RRAM device *without* the HfO_2 is close to 6 kΩ, which is only a fraction of HRS resistance. This strongly indicates that the series resistance R_{series} (i.e. $R_{sh,G}$ + R_c) of GS-RRAM is not the major factor that contributes to the increases of the HRS resistance in GS-RRAM. On the contrary, this outcome suggests that the difference between the R_{switch} of GS-RRAM ($R_{int,G}$ + $R_{filament,G}$) and Pt-RRAM ($R_{int,Pt}$ + $R_{filament,Pt}$) determines the HRS of GS-RRAM and Pt-RRAM, respectively.

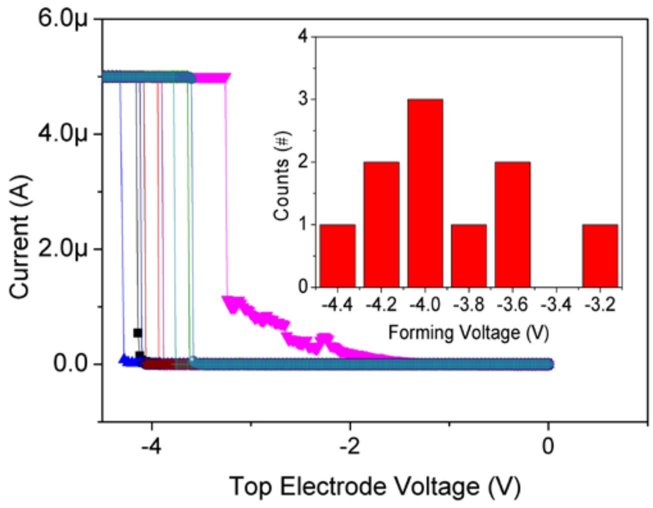

Supplementary Figure 9 Forming of GS-RRAM. The top electrode is the TiN electrode during the forming process. Forming curves are collected from 10 cells with 5μA compliance current. Inset: forming voltage distribution.

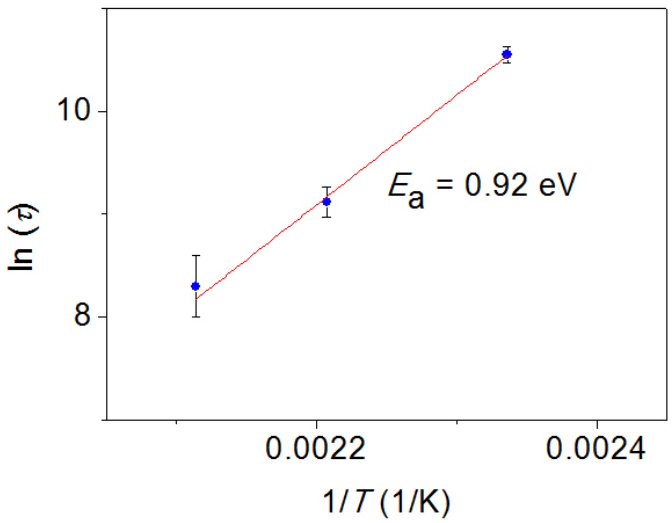

Supplementary Figure 10 Arrhenius type plot of the wait time versus 1/*T* extracted from Fig. 4b. The sudden transitions to the OFF state in Fig. 4b corresponds to the rupture of the oxygen vacancy based filament by the diffusion of oxygen ion towards HfO_x layer. The activation energy of the barriers can be extracted from temperature dependence of the characteristic dwell time for RESET transition (Arrhenius equation in Methods). From the linear fitting of retention time in logarithm scale versus reciprocal temperature, we estimate of the activation energy E_a for ion migration in graphene to be 0.92 eV.

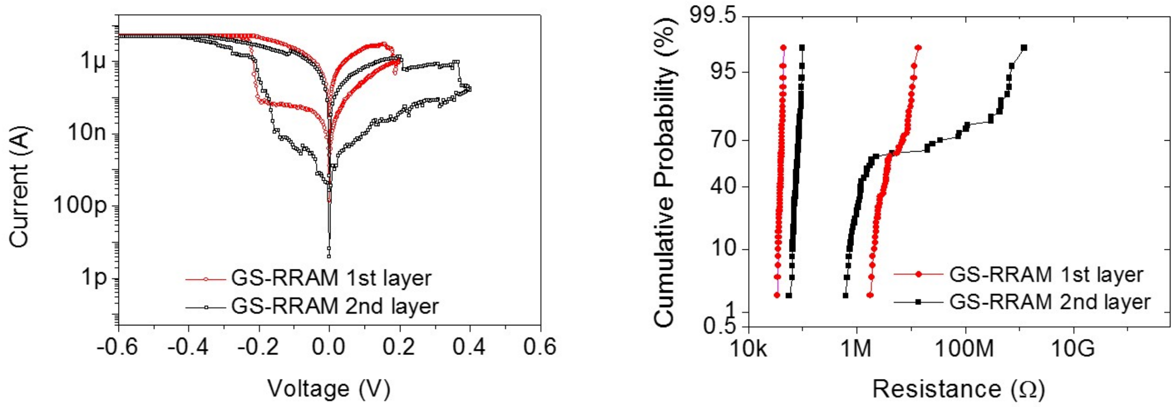

Supplementary Figure 11 Typical DC I-V switching and HRS/LRS characteristics of bottom layer and top layer of GS-RRAM. The GS-RRAM in the bottom layer exhibited even lower RESET current with similar SET voltages. However, there were some discrepancies in the RESET voltages for bottom and top layer. Importantly, the overall RESET power is still similar due to lower RESET current. Qualitatively similar memory windows were observed for top and bottom devices. The lowest memory window in the second layer is still above 10×.

Supplementary Table

	Thickness in nm (Electrode+SiO_2)	F = 20 nm	F = 22 nm	F = 26 nm
Etch ratio 30 Etch Angle 88°	11nm (Pt=5nm)	54	60	70
	16nm (Pt=10nm)	37	41	48
	21nm (Pt=15nm)	28	31	37
Etch Angle 89°	6.3nm (graphene=0.3nm)	181	200	236

Supplementary Table 1 Analysis of the number of achievable stacks with a dielectric thickness of 6nm. The achievable number of stacks can be calculated using the equation for reliability projection from reference [88]. Total stack height = $R\times F/T$ (R is the etching aspect ratio, F is the lithographic half pitch, and T is the combined thickness of the plane electrode and the dielectric in between). Assuming SiO_2 thickness of 6 nm, half-pitch of 22nm, and etch angle increase of just 1°, the maximum graphene RRAM stacks possible will be 200 stacks compared to the 60 stacks possible with Pt-RRAM. With an operating voltage of 0.2V in our GS-RRAM and a higher etching angle, we expect the number of possible graphene RRAM stacks to increase even more since a thinner dielectric can be used.

Supplementary Notes

Supplementary Note 1 3D vertical cross-point architectures

A pressing imperative for RRAM technology is to adopt a bit-cost-effective 3D architecture satisfying the requirements of performance metrics (density, latency, and energy consumption), which surpass those of 3D stackable multi-bit NAND Flash technology. Many industry/research groups [89-93] are actively working on variations of 3D vertical cross-point architectures as shown in Supplementary Fig. S1. The graphene RRAM in this work (with pillar electrode and planar graphene electrode) is compatible with all the 3D vertical cross-point architectures recently introduced [89-93].

The integration density of such 3D architectures depends on the number of stacks which is limited by the plane electrode thickness, the sheet resistance of the plane electrode, the dielectric thickness (related to the programming voltages and cross-talk), the pillar etch angle, the lithographic pitch, and the resistance of the pillar/plane electrode [88,94].

Since the total pillar height is limited, a thin device structure will be important for ultra-high density storage[88,94]. However, there is a fundamental limitation on how thin the metal plane electrode can be.

There has been a recent report of an RRAM structure with a sub-5nm thick vertical TiN electrode [95]. Although it is possible to form such sub-5nm metal electrodes, the main challenge lies not in the thickness of the metal, but in the high sheet resistance. All metal films are known to exhibit a steep exponential increase in sheet resistance as the thickness decreases under 10 nm[88,96]. This is because extremely thin metal films tend to form discontinuous islands, and thin dielectric layers are formed on the grain boundaries[96]. Such high sheet resistance of the plane electrode will result in a significant voltage drop on the electrode and severely degrade the write/read margin of the 3D RRAM structure[88,94], which limits the integration density. Hence, producing a sub-5nm conducting film with a low enough sheet resistance for 3D RRAM is a difficult task without using special methods or materials.

Graphene's sheet resistance per thickness is significantly lower than that of any metal. Graphene has been experimentally proven through the use of doping technique[97] to have sheet resistance as low as 125 - 200 Ω per square[1,86,97] with a monolayer thickness. These levels of resistance are something impossible to achieve (at such thickness) with conventional metal. From the measurements, graphene exhibited superior sheet resistance value per thickness (i.e. graphene is 20× thinner and 12× more resistive) compared to Pt after fabrication (Supplementary Section 7). Considering the nonlinear increase of Pt sheet resistance in such a scale, the actual sheet resistance of Pt when it is as thin as graphene will be drastically higher.

It is also important to note that metal contact to graphene is an ohmic contact, and the contact resistance is relatively low due to the graphene's semi-metallic nature[97]. An optimized

metal/graphene specific contact resistivity is 7.5×10^{-8} Ω cm^2 [98]. This value is smaller than that of both Al and Pt contact to degenerately N-doped silicon ($2\times10^{20}cm^{-3}$) as shown in [99].

From the analysis in the previous work[88], the required dielectric thickness is approximately 6 nm of SiO_2 in between each layer if the devices are to work with operating voltages of 3V (much higher than the 0.2V required for our GS-RRAM). The 6 nm SiO_2 is required since it can maintain a lifetime > 10 years at the operating voltage of 3V based on the breakdown voltage and the time dependent dielectric breakdown (TDDB) lifetime extrapolation for PECVD SiO_2 sandwiched between metal electrodes[100]. Finally, graphene (3Å) is significantly easier to etch vertically than Pt (6nm) during pillar formation. (Graphene is simply etched with weak O_2 plasma treatment.) This property is highly beneficial since the etch angle is a very important factor that determines the number of achievable stacks [88,94].

Supplementary Note 2 Comparison of using graphene as an oxygen detector (previous work, ref[82]) and for oxygen storage (this work)

In RRAM devices, the resistive switching is attributed to the formation (SET) and the subsequent rupture (RESET) of nanoscale conductive filaments involving oxygen ion migration[14,101-106]. A generally accepted theory claims that the filament formation is based on the oxygen ion movement from the switching material.

It is fairly well known that the oxygen function as dopants in graphene, and the doping level of graphene can be observed with Raman spectroscopy[82,107,108]. We have previously monitored the oxygen ion in a RRAM structure by inserting graphene film between the TiN layer and HfO_x[82].

The memory structures in our previous work and the current work are very different. In the previous work, the SET electrode is the TiN and the RESET electrode is the Pt. In our work, the SET electrode is the graphene edge and the RESET electrode is the TiN. Also the previous work is a planar structure and the current work is a vertical structure.

Although both previous and current work report low power consumption, the mechanisms for achieving low power consumption are fundamentally different. In the previous work, the low power was due to reduced RESET current from the high built-in series resistance of inter-layer graphene. The overall SET/RESET voltages (~2V) have few differences between structures “with” and “without” graphene interlayer.

In the current work, we see a drastic difference in SET/RESET voltages between GS-RRAM (~0.2V) and the Pt-based device (~1.5 to 2V). This is because the graphene, instead of the TiN layer, is used as the SET electrode. Here we are using graphene as a stand-alone oxygen reservoir, unlike in the previous work. The lowering of SET/RESET voltage is related to the lack of a TiO_xN_{1-x} barrier layer in the HfOx/graphene interface, and the ease of oxygen diffusion across the graphene electrode as explained in the main text.

Supplementary References

1 Lee, S., Lee, K., Liu, C.-H. & Zhong, Z. Homogeneous bilayer graphene film based flexible transparent conductor. *Nanoscale* **4**, 639-644 (2012).

2 Lee, S., Lee, K. & Zhong, Z. Wafer scale homogeneous bilayer graphene films by chemical vapor deposition. *Nano Lett.* **10**, 4702-4707 (2010).

3 Lee, S., Lee, K., Liu, C.-H., Kulkarni, G. S. & Zhong, Z. Flexible and transparent all-graphene circuits for quaternary digital modulations. *Nat. Commun.* **3**, 1018 (2012).

4 Lucchese, M. M. *et al.* Quantifying ion-induced defects and Raman relaxation length in graphene. *Carbon* **48**, 1592-1597 (2010).

5 Lee, H. *et al.* Low power and high speed bipolar switching with a thin reactive Ti buffer layer in robust HfO_2 based RRAM. in *Electron Devices Meeting, IEEE International.* 1-4 (2008).

6 Lee, H.-Y. *et al.* Low-power switching of nonvolatile resistive memory using hafnium oxide. *Jpn. J. Appl. Phys.* **46**, 2175 (2007).

7 Cheng, C. H., Yeh, F. S. & Chin, A. Low-Power High-Performance Non-Volatile Memory on a Flexible Substrate with Excellent Endurance. *Adv. Mater.* **23**, 902-905 (2011).

8 Wu, M.-C., Lin, Y.-W., Jang, W.-Y., Lin, C.-H. & Tseng, T.-Y. Low-power and highly reliable multilevel operation in 1T1R RRAM. *IEEE Electron Device Lett.* **32**, 1026-1028 (2011).

9 Wu, Y., Lee, B. & Wong, H.-S. Ultra-low power Al_2O_3-based RRAM with 1μA reset current. in *VLSI Technology Systems and Applications, International Symposium on.* 136-137 (2010).

10 Goux, L., Lisoni, J. G., Wang, X. P., Jurczak, M. & Wouters, D. J. Optimized Ni oxidation in 80-nm contact holes for integration of forming-free and low-power Ni/NiO/Ni memory cells. *Electron Devices, IEEE Transactions on* **56**, 2363-2368 (2009).

11 Tsunoda, K. *et al.* Low power and high speed switching of Ti-doped NiO ReRAM under the unipolar voltage source of less than 3 V. in *Electron Devices Meeting, IEEE International.* 767-770 (2007).

12 Hou, T.-H. *et al.* Evolution of RESET current and filament morphology in low-power HfO2 unipolar resistive switching memory. *Appl. Phys. Lett.* **98**, 103511 (2011).

13 Lee, H. *et al.* Low-power and nanosecond switching in robust hafnium oxide resistive memory with a thin Ti cap. *IEEE Electron Device Lett.* **31**, 44-46 (2010).

14 Wong, H.-S. P. *et al.* Metal–oxide RRAM. *Proc. IEEE* **100**, 1951-1970 (2012).

15 Wang, J.-C. *et al.* Low-power and high-reliability gadolinium oxide resistive switching memory with remote ammonia plasma treatment. *Jpn. J. Appl. Phys.* **52**, 04CD07 (2013).

16 Bai, Y. *et al.* Low power W: AlO_x/WO_x bilayer resistive switching structure based on conductive filament formation and rupture mechanism. *Appl. Phys. Lett.* **102**, 173503 (2013).

17 Yang, L. *et al.* Linear scaling of reset current down to 22-nm node for a novel RRAM. *IEEE Electron Device Lett.* **33**, 89-91 (2012).

18 Shima, H. *et al.* Voltage polarity dependent low-power and high-speed resistance switching in CoO resistance random access memory with Ta electrode. *Appl. Phys. Lett.* **93**, 113504 (2008).

19 Cheng, C. *et al.* Highly uniform low-power resistive memory using nitrogen-doped tantalum pentoxide. *Solid-State Electron.* **73**, 60-63 (2012).

20 Raghavan, N. *et al.* Very low reset current for an RRAM device achieved in the oxygen-vacancy-controlled regime. *IEEE Electron Device Lett.* **32**, 716-718 (2011).

21 Walczyk, C. *et al.* Pulse-induced low-power resistive switching in HfO_2 metal-insulator-metal diodes for nonvolatile memory applications. *Journal of Applied Physics* **105**, 114103-114103-114106 (2009).

22 Cheng, C., Chin, A. & Yeh, F. Novel ultra-low power RRAM with good endurance and retention. in *VLSI Technology, Symposium on.* 85-86 (2010).

23 Wu, Y., Yu, S., Lee, B. & Wong, P. Low-power $TiN/Al_2O_3/Pt$ resistive switching device with sub-20 μA switching current and gradual resistance modulation. *J. Appl. Phys.* **110**, 094104 (2011).

24 Baek, I. *et al.* Highly scalable nonvolatile resistive memory using simple binary oxide driven by asymmetric unipolar voltage pulses. in *Electron Devices Meeting, IEEE International.* 587-590 (2004).

25 Chen, A. *et al.* Non-volatile resistive switching for advanced memory applications. in *Electron Devices Meeting, IEEE International.* 746-749 (2005).

26 Wei, Z. *et al.* Highly reliable TaOx ReRAM and direct evidence of redox reaction mechanism. in *Electron Devices Meeting, IEEE International.* 1-4 (2008).

27 Tseng, Y. H., Huang, C.-E., Kuo, C., Chih, Y.-D. & Lin, C. J. High density and ultra small cell size of contact ReRAM (CR-RAM) in 90nm CMOS logic technology and circuits. in *Electron Devices Meeting (IEDM), 2009 IEEE International.* 1-4 (2009).
28 Wang, X. *et al.* Highly compact 1T-1R architecture ($4F^2$ footprint) involving fully CMOS compatible vertical GAA nano-pillar transistors and oxide-based RRAM cells exhibiting excellent NVM properties and ultra-low power operation. in *Electron Devices Meeting (IEDM), 2012 IEEE International.* 20.26. 21-20.26. 24 (2012).
29 Shen, W. C. *et al.* High-K metal gate contact RRAM (CRRAM) in pure 28nm CMOS logic process. in *Electron Devices Meeting (IEDM), 2012 IEEE International.* 31.36. 31-31.36. 34 (2012).
30 Kim, S. *et al.* Ultrathin (<10nm) Nb_2O_5/NbO_2 hybrid memory with both memory and selector characteristics for high density 3D vertically stackable RRAM applications. in *VLSI Technology (VLSIT), 2012 Symposium on.* 155-156 (2012).
31 Chen, H.-Y. *et al.* HfO_x based vertical resistive random access memory for cost-effective 3D cross-point architecture without cell selector. in *Electron Devices Meeting (IEDM), 2012 IEEE International.* 20.27. 21-20.27. 24 (2012).
32 Wu, Y. *et al.* First Demonstration of RRAM Patterned by Block Copolymer Self-Assembly. in *Electron Devices Meeting (IEDM), 2013 IEEE International.* 1-20.28 (2013).
33 Li, K.-S. *et al.* Utilizing Sub-5 nm sidewall electrode technology for atomic-scale resistive memory fabrication. in *VLSI Technology (VLSI-Technology): Digest of Technical Papers, 2014 Symposium on.* 1-2 (2014).
34 Sohn, J., Lee, S., Jiang, Z., Chen, H.-Y. & Wong, H.-S. P. Atomically thin graphene plane electrode for 3D RRAM. in *Electron Devices Meeting (IEDM), 2014 IEEE International.* 5.3. 1-5.3. 4 (2014).
35 Sekar, D. *et al.* Technology and circuit optimization of resistive RAM for low-power, reproducible operation. in *Electron Devices Meeting (IEDM), 2014 IEEE International.* 28.23. 21-28.23. 24 (2014).
36 Jo, S. H., Kumar, T., Narayanan, S., Lu, W. D. & Nazarian, H. 3D-stackable crossbar resistive memory based on field assisted superlinear threshold (FAST) selector. in *Electron Devices Meeting (IEDM), 2014 IEEE International.* 6.7. 1-6.7. 4 (2014).
37 Lee, B. & Wong, H.-S. NiO resistance change memory with a novel structure for 3D integration and improved confinement of conduction path. in *VLSI Technology, 2009 Symposium on.* 28-29 (2009).
38 Chen, Y. *et al.* Highly scalable hafnium oxide memory with improvements of resistive distribution and read disturb immunity. in *Electron Devices Meeting (IEDM), 2009 IEEE International.* 1-4 (2009).
39 Sakotsubo, Y. *et al.* A new approach for improving operating margin of unipolar ReRAM using local minimu m of reset voltage. in *VLSI Technology (VLSIT), 2010 Symposium on.* 87-88 (2010).
40 Ho, C. *et al.* 9nm half-pitch functional resistive memory cell with <1µA programming current using thermally oxidized sub-stoichiometric WO_x film. in *Electron Devices Meeting (IEDM), 2010 IEEE International.* 19.11. 11-19.11. 14 (2010).
41 Chien, W. *et al.* A forming-free WO_x resistive memory using a novel self-aligned field enhancement feature with excellent reliability and scalability. in *Electron Devices Meeting (IEDM), 2010 IEEE International.* 19.12. 11-19.12. 14 (2010).
42 Cheng, C., Tsai, C., Chin, A. & Yeh, F. High performance ultra-low energy RRAM with good retention and endurance. in *Electron Devices Meeting (IEDM), 2010 IEEE International.* 19.14. 11-19.14. 14 (2010).
43 Lee, J. *et al.* Diode-less nano-scale ZrO_x/HfO_x RRAM device with excellent switching uniformity and reliability for high-density cross-point memory applications. in *Electron Devices Meeting (IEDM), 2010 IEEE International.* 19.15. 11-19.15. 14 (2010).
44 Tran, X. *et al.* High performance unipolar $AlO_y/HfO_x/Ni$ based RRAM compatible with Si diodes for 3D application. in *VLSI Technology (VLSIT), 2011 Symposium on.* 44-45 (2011).
45 Yi, J. *et al.* Highly reliable and fast nonvolatile hybrid switching ReRAM memory using thin Al_2O_3 demonstrated at 54nm memory array. in *VLSI Technology (VLSIT), 2011 Symposium on.* 48-49 (2011).
46 Kim, Y.-B. *et al.* Bi-layered RRAM with unlimited endurance and extremely uniform switching. in *VLSI Technology (VLSIT), 2011 Symposium on.* 52-53 (2011).
47 Govoreanu, B. *et al.* $10\times10nm^2$ Hf/HfO $_x$ crossbar resistive RAM with excellent performance, reliability and low-energy operation. in *Electron Devices Meeting (IEDM), 2011 IEEE International.* 31.36. 31-31.36. 34 (2011).
48 Aratani, K. *et al.* A novel resistance memory with high scalability and nanosecond switching. in *Electron Devices Meeting, 2007. IEDM 2007. IEEE International.* 783-786 (2007).

49 Sakamoto, T. *et al.* A Ta_2O_5 solid-electrolyte switch with improved reliability. in *VLSI Technology, 2007 IEEE Symposium on.* 38-39 (2007).
50 Lin, Y.-Y. *et al.* A novel TiTe buffered Cu-GeSbTe/SiO_2 electrochemical resistive memory (ReRAM). in *VLSI Technology (VLSIT), 2010 Symposium on.* 91-92 (2010).
51 Wang, M. *et al.* A novel Cu_xSi_yO resistive memory in logic technology with excellent data retention and resistance distribution for embedded applications. in *VLSI Technology (VLSIT), 2010 Symposium on.* 89-90 (2010).
52 Vianello, E. *et al.* Sb-doped GeS_2 as performance and reliability booster in Conductive Bridge RAM. in *Electron Devices Meeting (IEDM), 2012 IEEE International.* 31.35. 31-31.35. 34 (2012).
53 Goux, L. *et al.* Field-driven ultrafast sub-ns programming in W\Al_2O_3\Ti\CuTe-based 1T1R CBRAM system. in *VLSI Technology (VLSIT), 2012 Symposium on.* 69-70 (2012).
54 Guy, J. *et al.* Investigation of the physical mechanisms governing data-retention in down to 10nm Nano-trench Al_2O_3/CuTeGe Conductive Bridge RAM (CBRAM). in *Proc. IEEE IEDM.* (2013).
55 Vianello, E. *et al.* Resistive memories for ultra-low-power embedded computing design. in *Electron Devices Meeting (IEDM), 2014 IEEE International.* 6.3. 1-6.3. 4 (2014).
56 Sills, S. *et al.* A copper ReRAM cell for Storage Class Memory applications. in *VLSI Technology (VLSI-Technology): Digest of Technical Papers, 2014 Symposium on.* 1-2 (2014).
57 Yi, J. *et al.* Novel cell structure of PRAM with thin metal layer inserted GeSbTe. in *Electron Devices Meeting, 2003. IEDM'03 Technical Digest. IEEE International.* 37.33. 31-37.33. 34 (2003).
58 Pirovano, A. *et al.* Scaling analysis of phase-change memory technology. in *Electron Devices Meeting, 2003. IEDM'03 Technical Digest. IEEE International.* 29.26. 21-29.26. 24 (2003).
59 Matsui, Y. *et al.* Ta_2O_5 interfacial layer between GST and W plug enabling low power operation of phase change memories. in *Electron Devices Meeting, 2006. IEDM'06. International.* 1-4 (2006).
60 Mantegazza, D., Ielmini, D., Varesi, E., Pirovano, A. & Lacaita, A. Statistical analysis and modeling of programming and retention in PCM arrays. in *Electron Devices Meeting, 2007. IEDM 2007. IEEE International.* 311-314 (2007).
61 Chen, W. *et al.* A novel cross-spacer phase change memory with ultra-small lithography independent contact area. in *Electron Devices Meeting, 2007. IEDM 2007. IEEE International.* 319-322 (2007).
62 Annunziata, R. *et al.* Phase change memory technology for embedded non volatile memory applications for 90nm and beyond. in *Electron Devices Meeting (IEDM), 2009 IEEE International.* 1-4 (2009).
63 Fantini, A. *et al.* N-doped GeTe as performance booster for embedded phase-change memories. in *Electron Devices Meeting (IEDM), 2010 IEEE International.* 29.21. 21-29.21. 24 (2010).
64 Lee, S. *et al.* Highly productive PCRAM technology platform and full chip operation: Based on $4F^2$ (84nm pitch) cell scheme for 1 Gb and beyond. in *Electron Devices Meeting (IEDM), 2011 IEEE International.* 3.3. 1-3.3. 4 (2011).
65 Cheng, H. *et al.* A high performance phase change memory with fast switching speed and high temperature retention by engineering the $Ge_xSb_yTe_z$ phase change material. in *Electron Devices Meeting (IEDM), 2011 IEEE International.* 3.4. 1-3.4. 4 (2011).
66 Morikawa, T. *et al.* A low power phase change memory using low thermal conductive doped-$Ge_2Sb_2Te_5$ with nano-crystalline structure. in *Electron Devices Meeting (IEDM), 2012 IEEE International.* 31.34. 31-31.34. 34 (2012).
67 Cheng, H. *et al.* Atomic-level enginereeing of phase change material for novel fast-switching and high-endurance PCM for storage class memory application. *IEDM Tech. Dig*, 758-761 (2013).
68 Ha, Y. *et al.* An edge contact type cell for phase change RAM featuring very low power consumption. in *VLSI Technology, 2003. Digest of Technical Papers. 2003 Symposium on.* 175-176 (2003).
69 Pellizzer, F. *et al.* Novel μtrench phase-change memory cell for embedded and stand-alone non-volatile memory applications. in *VLSI Technology, 2004. Digest of Technical Papers. 2004 Symposium on.* 18-19 (2004).
70 Breitwisch, M. *et al.* Novel lithography-independent pore phase change memory. in *VLSI Technology, 2007 IEEE Symposium on.* 100-101 (2007).
71 Kim, I. *et al.* High performance PRAM cell scalable to sub-20nm technology with below $4F^2$ cell size, extendable to DRAM applications. in *VLSI Technology (VLSIT), 2010 Symposium on.* 203-204 (2010).
72 Liang, J., Jeyasingh, R. G. D., Chen, H.-Y. & Wong, H.-S. A 1.4μA reset current phase change memory cell with integrated carbon nanotube electrodes for cross-point memory application. in *VLSI Technology (VLSIT), 2011 Symposium on.* 100-101 (2011).

73 Bedeschi, F. *et al.* An 8Mb demonstrator for high-density 1.8 V phase-change memories. in *VLSI Circuits, 2004. Digest of Technical Papers. 2004 Symposium on.* 442-445 (2004).
74 Oh, H. Enhanced write performance of a 64Mb phase-change random access memory. in *IEEE International Solid State Circuits Conference.* **1**, 48-584 (2005).
75 Kang, S. *et al.* A 0.1μm 1.8V 256Mb 66Mhz synchronous burst pram. in *2006 IEEE International Solid State Circuits Conference-Digest of Technical Papers.* 487-496 (2006).
76 Hanzawa, S. *et al.* A 512kB embedded phase change memory with 416kB/s write throughput at 100μA cell write current. in *Solid-State Circuits Conference, 2007. ISSCC 2007. Digest of Technical Papers. IEEE International.* 474-616 (2007).
77 Gajek, M. *et al.* Spin torque switching of 20 nm magnetic tunnel junctions with perpendicular anisotropy. *Appl. Phys. Lett.* **100**, 132408 (2012).
78 Sato, H. *et al.* Properties of magnetic tunnel junctions with a MgO/CoFeB/Ta/CoFeB/MgO recording structure down to junction diameter of 11 nm. *Appl. Phys. Lett.* **105**, 062403 (2014).
79 Wu, N. *et al.* Synthesis of network reduced graphene oxide in polystyrene matrix by a two-step reduction method for superior conductivity of the composite. *J. Mater. Chem.* **22**, 17254-17261 (2012).
80 Lehtinen, O., Kurasch, S., Krasheninnikov, A. & Kaiser, U. Atomic scale study of the life cycle of a dislocation in graphene from birth to annihilation. *Nat. Commun.* **4** (2013).
81 Kurasch, S. *et al.* Atom-by-atom observation of grain boundary migration in graphene. *Nano Lett.* **12**, 3168-3173 (2012).
82 Tian, H. *et al.* Monitoring Oxygen Movement by Raman Spectroscopy of Resistive Random Access Memory with a Graphene-Inserted Electrode. *Nano Lett.* **13**, 651-657 (2013).
83 Klootwijk, J. & Timmering, C. Merits and limitations of circular TLM structures for contact resistance determination for novel III-V HBTs. in *2004 international conference on microelectronic test structures.* 247-252 (2004).
84 Wang, Q. H., Kalantar-Zadeh, K., Kis, A., Coleman, J. N. & Strano, M. S. Electronics and optoelectronics of two-dimensional transition metal dichalcogenides. *Nat. Nanotechnol.* **7**, 699-712 (2012).
85 Bonaccorso, F., Sun, Z., Hasan, T. & Ferrari, A. Graphene photonics and optoelectronics. *Nat. Photonics* **4**, 611-622 (2010).
86 Wang, Y., Tong, S. W., Xu, X. F., Özyilmaz, B. & Loh, K. P. Interface Engineering of Layer‐by‐Layer Stacked Graphene Anodes for High‐Performance Organic Solar Cells. *Adv. Mater.* **23**, 1514-1518 (2011).
87 Blake, P. *et al.* Graphene-based liquid crystal device. *Nano Lett.* **8**, 1704-1708 (2008).
88 Chen, H.-Y. *et al.* Experimental study of plane electrode thickness scaling for 3D vertical resistive random access memory. *Nanotechnology* **24**, 465201 (2013).
89 Yu, S., Chen, H.-Y., Gao, B., Kang, J. & Wong, H.-S. P. HfO_x-based vertical resistive switching random access memory suitable for bit-cost-effective three-dimensional cross-point architecture. *ACS nano* **7**, 2320-2325 (2013).
90 Cha, E. *et al.* Nanoscale (~10nm) 3D vertical RRAM and NbO_2 threshold selector with TiN electrode. in *Electron Devices Meeting (IEDM), 2013 IEEE International.* 10.15. 11-10.15. 14 (2013).
91 Deng, Y. *et al.* Design and Optimization Methodology for 3D RRAM Arrays. in *IEEE International Electron Devices Meeting (IEDM).* 9-11 (2013).
92 Hsu, C.-W. *et al.* 3D Vertical TaO_x/TiO_2 RRAM with over 10^3 Self-Rectifying Ratio and Sub-μA Operating Current. *IEEE Int. Electron Devices Meet. Tech. Dig*, 10-13 (2013).
93 Cha, E. *et al.* Nanoscale (10nm) 3d vertical reram and Nbo_2 threshold selector with tin electrode. in *Proceedings of the IEEE International Electron Devices Meeting (IEDM).* 10.15 (2013).
94 Yu, S. *et al.* 3D vertical RRAM-Scaling limit analysis and demonstration of 3D array operation. *VLSI Technology (VLSIT), 2013 Symposium on*, T158-T159 (2013).
95 Li, K.-S. *et al.* Utilizing sub-5 nm sidewall electrode technology for atomic-scale resistive memory fabrication. in *Symp. VLSI Techology.* p131-132 2014.
96 Zhigal'skii, G. P. & Jones, B. K. *The Physical Properties of Thin Metal Films*. (Taylor & Francis, 2003).
97 Bae, S. *et al.* Roll-to-roll production of 30-inch graphene films for transparent electrodes. *Nat. Nanotechnol.* **5**, 574-578 (2010).
98 Robinson, J. A. *et al.* Contacting graphene. *Appl. Phys. Lett.* **98**, 053103 (2011).
99 Yu, A. Electron tunneling and contact resistance of metal-silicon contact barriers. *Solid-State Electron.* **13**, 239-247 (1970).
100 Zhang, G., Hu, C., Yu, P., Chiang, S. & Hamdy, E. Metal-to-metal antifuses with very thin silicon dioxide films. *IEEE Electron Device Lett.* **15**, 310-312 (1994).

101 Guan, X., Yu, S. & Wong, H.-S. On the switching parameter variation of metal-oxide RRAM—Part I: Physical modeling and simulation methodology. *Electron Devices, IEEE Transactions on* **59**, 1172-1182 (2012).
102 Yu, S., Guan, X. & Wong, H.-S. On the switching parameter variation of metal oxide RRAM—Part II: Model corroboration and device design strategy. *Electron Devices, IEEE Transactions on* **59**, 1183-1188 (2012).
103 Yang, Y. *et al.* Observation of conducting filament growth in nanoscale resistive memories. *Nat. Commun.* **3**, 732 (2012).
104 Kwon, D.-H. *et al.* Atomic structure of conducting nanofilaments in TiO_2 resistive switching memory. *Nat. Nanotechnol.* **5**, 148-153 (2010).
105 Lee, M.-J. *et al.* Electrical manipulation of nanofilaments in transition-metal oxides for resistance-based memory. *Nano Lett.* **9**, 1476-1481 (2009).
106 Waser, R. & Aono, M. Nanoionics-based resistive switching memories. *Nat. Mater.* **6**, 833-840 (2007).
107 Das, A. *et al.* Monitoring dopants by Raman scattering in an electrochemically top-gated graphene transistor. *Nat. Nanotechnol.* **3**, 210-215 (2008).
108 Tongay, S. *et al.* Rectification at graphene-semiconductor interfaces: zero-gap semiconductor-based diodes. *Physical Review X* **2**, 011002 (2012).
